# The hard X-ray ptychography endstation at the Taiwan Photon Source

**DOI:** 10.1107/S1600577526001591

**Published:** 2026-03-20

**Authors:** Yi-Wei Tsai, Gung-Chian Yin, Bo-Yi Chen, Hsin-Wei Chen, Kang-Ching Chu, Chi-Yuan Cheng, Po-Wei Lee, Jhih-Min Lin, Chun-Yu Chen, Yu-Shan Huang

**Affiliations:** a National Synchrotron Radiation Research Center Hsinchu300 Taiwan; RIKEN SPring-8 Center, Japan

**Keywords:** ptychography, syn­chrotron, nano-imaging, coherent X-ray scattering

## Abstract

Following our initial demonstration of ptychography at the Taiwan Photon Source (TPS), the system has been substantially upgraded. This article details the design and performance of the new hard X-ray 2D ptychography endstation, TPS 25A2. A zone-plate focusing system has been inserted downstream of the previous Kirkpatrick–Baez mirror optics at the TPS 25A2 beamline. Operating at a photon energy of 8.979 keV, the endstation routinely achieves a spatial resolution of sub-10 nm in 2D, with a highest achieved resolution of 6.9 nm on a standard Siemens star test pattern. We present the full instrumental specifications and performance benchmarks, and showcase the endstation’s capabilities with imaging examples of inorganic nanoparticles and a complex biological structure. The establishment of TPS 25A2 pro­vides the scientific community with a powerful and accessible tool for nanoscale imaging.

## Abstract 2

The newly commissioned hard X-ray ptychography endstation at the Taiwan Photon Source, TPS 25A2, is introduced, detailing the instrumental specifications and demonstrating a benchmark spatial resolution of 6.9 nm in 2D, showcased through the imaging of a standard test pattern, inorganic nanoparticles and a complex biological structure.

## 1. Introduction

Ptychography, an advanced lensless imaging technique, pro­vides high spatial resolution beyond conventional X-ray microscopy (Pfeiffer, 2018 ▸). Fundamentally, ptychography is a scanning-based variant of coherent diffraction imaging (CDI). The CDI method involves illuminating a sample with a coherent light source, recording the resulting far-field diffraction pattern and using iterative phase retrieval algorithms to reconstruct an image of the sample. The advantages and challenges of CDI are clear. The primary advantage is that, as a lensless method, its spatial resolution is not limited by the quality of imaging optics. This is particularly important in the hard X-ray regime, where high-quality lenses are difficult to manufacture and often imperfect (Nazaretski *et al.*, 2022 ▸; Kewish *et al.*, 2024 ▸). The main challenge, however, is the strict requirement for coherence. CDI demands a nearly fully coherent source, which was difficult to achieve in the hard X-ray regime until the advent of third-generation syn­chrotron radiation sources (Singer & Vartanyants, 2014 ▸) and free-electron laser (FEL) facilities (Huang *et al.*, 2021 ▸).

Since the first successful CDI demonstration in 1999 (Miao *et al.*, 1999 ▸), the associated algorithms have developed dra­mat­i­cal­ly. Early CDI experiments were limited not only by the need for a highly coherent source but also by the strict requirement for an isolated sample to pro­vide a real-space support constraint. In 2004, the proposal of the ptychographical iterative engine (PIE) (Rodenburg & Faulkner, 2004 ▸) ushered in the era of ptychography, a scanning-based CDI method that replaces the isolation constraint with a more flexible overlap constraint. The development of the extended PIE (ePIE) in 2009 (Maiden & Rodenburg, 2009 ▸) and difference map (DM) method (Thibault *et al.*, 2009 ▸), which co-reconstructs the illuminating probe and the sample, dra­mat­i­cal­ly improved image quality and relaxed the constraint of needing a perfectly known incident wavefield. Subsequently, alternative frameworks, such as the maximum likelihood (ML) method, was proposed in 2012 (Thibault & Guizar-Sicairos, 2012 ▸), followed by the crucial development of the mixture-state model in 2013 (Thibault & Menzel, 2013 ▸), which addressed the issue of partially coherent sources.

The field of ptychography continues to evolve rapidly. 3D reconstructions, such as ptychographic X-ray-computed tomography (PXCT) (Aidukas *et al.*, 2024 ▸) and ptychographic laminography (Holler *et al.*, 2019 ▸), high-throughput fly scan methods (Deng *et al.*, 2015 ▸; Jones *et al.*, 2022 ▸) and *in situ* (Grote *et al.*, 2022 ▸) and *operando* studies (Sasaki *et al.*, 2025 ▸), are active areas of research. Beyond ambient conditions, advanced sample environments, such as cryogenic (Holler *et al.*, 2018 ▸) and high-temperature (Holler *et al.*, 2022 ▸) stages, are expanding the capabilities of ptychography. Concurrently, the inherent measurement speed bottleneck of PXCT is being effectively overcome by leveraging sparse sampling strategies (Gao *et al.*, 2024 ▸). Furthermore, with fourth-generation syn­chrotron radiation facilities now in operation, the dramatic increase in coherent flux pro­vides ideal conditions for these advanced techniques (Li *et al.*, 2022 ▸; Bruno *et al.*, 2024 ▸; Chushkin & Zontone, 2025 ▸). The potential of ptychography in the near future is highly anticipated.

Ptychography offers a powerful and distinctive contrast mechanism. In conventional X-ray techniques, such as transmission microscopy, image contrast relies on the attenuation of X-rays as they pass through the sample. However, this absorption contrast is often inherently weak in the multi-keV X-ray energy range for thin specimens or those composed of light elements, making it difficult to visualize their internal structures. Ptychography overcomes this fundamental limitation by offering the quantitative retrieval of the X-ray phase shift passing through a sample, ϕ(**r**), which is more sensitive to variations in material density and composition than absorption in the hard X-ray regime.

For instance, for a 1 µm-thick chitin sample at 8.979 keV, the X-ray attenuation is merely 0.069%, and the induced phase shift is approximately 0.175 radians (10°) (Henke *et al.*, 1993 ▸; https://henke.lbl.gov/optical_constants/). While a 0.069% intensity variation is difficult to detect, a phase shift of this magnitude pro­vides strong clear contrast, making ptychography well suited for biological and weakly absorbing materials.

Furthermore, because ptychography is fundamentally a 2D transmission imaging technique, it can be readily integrated with other established X-ray methods. As mentioned, it can be extended into three dimensions by combining it with techniques such as tomography or laminography. It can also be coupled with other analytical methods to create multi-modal approaches. For instance, by simultaneously collecting signals such as X-ray fluorescence (XRF) (Deng *et al.*, 2017 ▸), crystal X-ray diffraction (XRD) (Grote *et al.*, 2023 ▸), X-ray absorption near-edge structure (XANES) (Uematsu *et al.*, 2021 ▸), *etc*., researchers can acquire chemical and structural information, respectively, which is co-registered with the high-resolution morphological image pro­vided by ptychography.

To contribute to this growing field and pro­vide these advanced capabilities to the scientific community, a preliminary study of mirror-based ptychography was performed, achieving a spatial resolution of sub-20 nm (Tsai *et al.*, 2021 ▸). Currently, we have established a zone-plate-based ptychography endstation in TPS 25A2 at the Taiwan Photon Source (TPS). This instrument serves a dual role: it not only pro­vides a powerful nanoscale imaging technique for general users but also serves as a platform for future developments in ptychography. In this article, we present the detailed design, technical specifications and commissioning results of the TPS 25A2 endstation.

## 2. Ptychography

**Figure 1:**
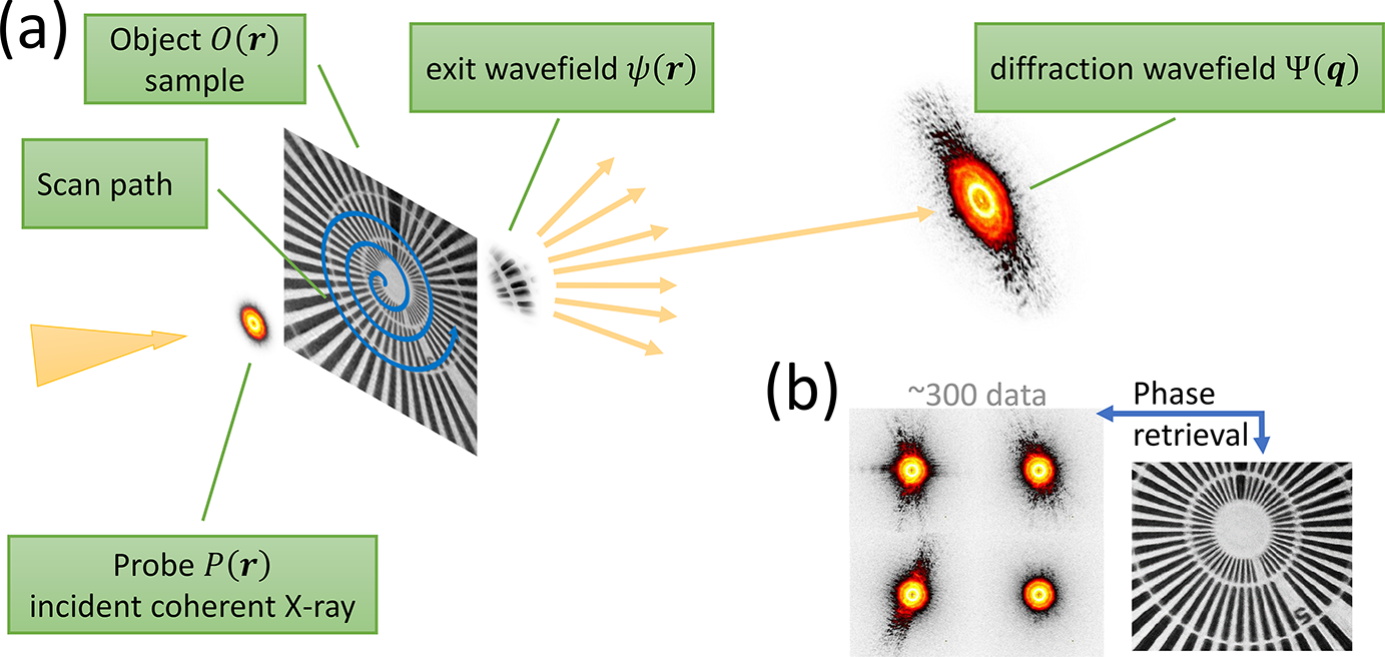
Schematic of ptychography.

In this section, a brief description of the fundamental principles of ptychography is pro­vided. The basic process is illustrated in Fig. 1 ▸. As shown in Fig. 1 ▸(*a*), a coherent incident wavefield, referred to as the probe *P*(**r**), illuminates a sample, or object, *O*(**r**). The wavefield immediately after the sample is the exit wave, ψ(**r**) = *P*(**r**)·*O*(**r**), which contains information about both the probe and the object. This exit wave propagates to the far field, forming a Fraunhofer diffraction pattern, *I*(**q**), which is recorded by an area detector. The recorded intensity is the squared modulus of the diffraction wavefield, Ψ(**q**), where **q** is the reciprocal space coordinate.

Theoretically, the exit wave ψ(**r**) and the diffraction wavefield Ψ(**q**) are related by a Fourier transform, Ψ(**q**) = 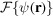. However, detectors can only record intensity, *I*(**q**) = |Ψ(**q**)|^2^, meaning the phase information of the diffraction wavefield is lost. To retrieve this lost phase, ptychography applies a scanning strategy that collects a series of diffraction patterns from overlapping regions on the sample [Fig. 1 ▸(*b*)]. This overlap creates informational redundancy, which is crucial for iterative algorithms to robustly solve for the object’s complex transmission function. Table 1 ▸ summarizes the key parameters and notations used in ptychography experiments.

The reconstructed complex-valued object function, *O*(**r**), pro­vides two distinct channels of information: amplitude and phase. It can be expressed as *O*(**r**) = 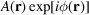, where *A*(**r**) is the amplitude and ϕ(**r**) is the phase shift.

In most applications, the phase component, ϕ(**r**), pro­vides the primary source of contrast. As mentioned previously, this phase information is particularly sensitive to variations in composition and density, especially for low-*Z* materials, where absorption contrast is minimal. The majority of ptychographic images presented in the literature and in this work are therefore phase images due to their superior signal-to-noise ratio and richer structural details.

The amplitude component, *A*(**r**), pro­vides complementary information related to the sample’s absorption and transmittance properties. The transmittance, *T*(**r**), defined as the ratio of transmitted to incident intensity, is equal to the squared amplitude: *T*(**r**) = |ψ(**r**)|^2^/|*P*(**r**)|^2^ = |*O*(**r**)|^2^ = *A*(**r**)^2^. The amplitude image *A*(**r**) is therefore equivalent to the square root of a conventional transmission image and pro­vides a direct measure of attenuation [1 − *T*(**r**)]. While less frequently used for morphological imaging, this quantitative amplitude information is crucial for advanced analytical techniques. Because it can be retrieved at each photon energy, it enables nano-X-ray absorption near-edge structure (nano-XANES) spectroscopy for elemental and chemical-state mapping.

## 3. The TPS 25A2 ptychography endstation

### 3.1. X-ray source and upstream optics

Ptychography experiments were performed at the TPS 25A2 beamline (Lin *et al.*, 2015 ▸) of the Taiwan Photon Source (Horiuchi, 2015 ▸). The TPS is a third-generation syn­chrotron radiation facility operating with a 3 GeV electron storage ring and a top-up injection current of 500 mA, and the TPS 25A2 beamline utilizes an in-vacuum undulator as its source.

**Figure 2:**
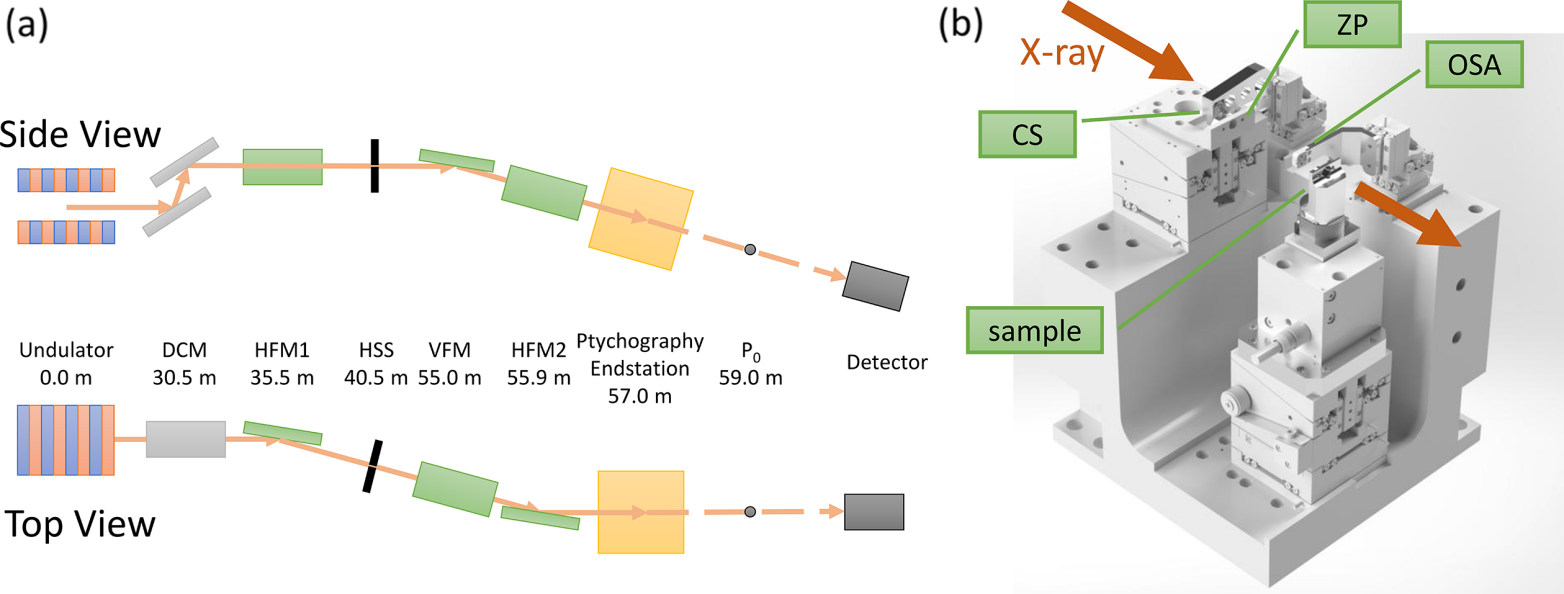
Schematic of the experimental setup. (*a*) The TPS 25A2 beamline and its upstream optics, and (*b*) the ptychography endstation.

As illustrated in Fig. 2 ▸(*a*), the pre-existing upstream optics consist of a Si(111) double-crystal monochromator (DCM), a set of precision slits acting as a secondary source, and an asymmetric three-mirror focusing system. Downstream of the DCM, the beam focusing is handled asymmetrically. In the horizontal direction, the beam is first focused by a horizontal focusing mirror (HFM1) onto the precision slits. These slits define a horizontal secondary source (HSS), which is subsequently re-focused by a second horizontal focusing mirror (HFM2). In the vertical direction, a single vertical focusing mirror (VFM) is utilized for focusing. The system is designed such that the horizontal and vertical focal planes are coincident at P_0_. These optical components collectively deliver a highly coherent X-ray beam to the downstream endstation in the energy range of 5 to 10 keV.

### 3.2. The ptychography endstation

The ptychography endstation, designated TPS 25A2, is located approximately 2 m upstream of the primary focal plane P_0_ of the TPS 25A2 optics. A schematic of the core components is shown in Fig. 2 ▸(*b*), including a central stop (CS), a zone plate (ZP), an order-sorting aperture (OSA) and stages for the optics and samples. The endstation is housed in a hutch where the ambient temperature is controlled to within ±0.05°C over one hour to ensure high mechanical stability.

#### 3.2.1. Zone-plate focusing optics

The nano-focusing optics for ptychography comprise a central stop (CS), a zone plate (ZP) and an order-sorting aperture (OSA). The central stop is a gold cylinder on a membrane with a diameter of 30 µm and a thickness of >50 µm (XRnanotech, Villigen, Switzerland). The primary focusing element is a gold ZP with a diameter of 100 µm, a thickness of >1300 nm and an outermost zone width of 56 nm (XRnanotech). By design, the focal length of the ZP is approximately 42 mm at 9 keV, and its calculated diffraction efficiency is approximately 27% at 9 keV, remaining above 20% in the energy range from 5.5 to 11 keV. The CS and OSA are mounted on a three-axis piezoelectric positioner (SLC-1720; SmarAct, Oldenburg, Germany), while the ZP is mounted on an XZ-stage (CLC-5252 for X and CHS-5237 for Z; SmarAct).

#### 3.2.2. Beam properties and coherence

To enhance spatial coherence for the experiments, the secondary source slits are typically set to an opening of 15 µm in the horizontal direction. The diameter of the zone plate was chosen to be smaller than the transverse coherence length. The resulting beam at the sample is therefore considered to be nearly fully coherent. The measured photon flux at the sample position under these conditions is approximately 5 × 10^8^ photons s^−1^ at 9 keV. The illuminated beam size on the sample can be varied from sub-100 nm to 3 µm by adjusting the sample’s defocus position. While a larger defocused beam significantly reduces the flux density and thus mitigates potential X-ray damage, it can slightly affect the ultimate spatial resolution. It should be noted that theoretically the attainable resolution is independent of the beam size; the observed dependence is attributed to temporary mechanical instabilities in the optical elements, which are currently being rectified. Furthermore, the maximum usable beam size is fundamentally limited by the CDI window of the reconstruction (Burdet *et al.*, 2014 ▸) (*W* in Table 1 ▸). To ensure an accurate reconstruction, the entire illuminated area on the sample must be contained within this window.

#### 3.2.3. Sample positioning and scanning

Samples are mounted on a positioning stack consisting of a three-axis long-range coarse stage (CLC-5252 for X/Y and CHS-5237 for Z; SmarAct) and a three-axis nanopositioning flexure stage (P-616; Physik Instrumente, Karlsruhe, Germany). The flexure stage pro­vides high-precision movements over a 100 µm range for the ptychography scan, while the coarse stage is used for sample navigation and for adjusting the illumination size by defocusing the sample along the optical axis.

#### 3.2.4. Detector systems

The endstation has been commissioned for operation at a photon energy of 8.979 ± 0.5 keV, near the Cu *K*-edge. The facility offers two detector configurations to collect the far-field coherent diffraction patterns. These consist of an Eiger 1M detector (Dectris, Baden-Daettwil, Switzerland) placed at a sample-to-detector distance (SDD) of 2.1 m, and an Eiger 16M (Dectris) at an SDD of 3.9 m. The pixel size for both detectors is 75 µm, and the pixel arrays are 1028 × 1062 and 4148 × 4362 for 1M and 16M, respectively. For typical ex­peri­ments, the Eiger 16M is operated in a 4M region-of-interest (ROI) mode, corresponding to a 2070 × 2167 pixel array.

#### 3.2.5. Control and data analysis

The control system of the endstation is based on a two-layer software architecture. Low-level communication with all hardware, including motors and detectors, is managed by the *Experimental Physics and Industrial Control System* (*EPICS*) (https://epics-controls.org). High-level experiment orchestration, including the execution of scan plans and data acquisition, is handled by the Python-based Bluesky framework (Allan *et al.*, 2019 ▸). For data processing, ptychographic re­con­struc­tions are performed using the GPU-accelerated *PyNX* toolkit (Favre-Nicolin *et al.*, 2020 ▸), supplemented by an in-house program for specialized analysis.

During data acquisition, the P-616 flexure stage is operated in a closed-loop feedback mode to ensure precise and repeatable sample positioning. To maintain maximum stability, all positioning stages for the upstream optical components and sample navigation are operated in open-loop mode to prevent any potential feedback-induced motion during a scan.

#### 3.2.6. Sample preparation

Currently, the TPS 25A2 endstation is configured for 2D imaging in a transmission geometry. In this configuration, the reconstructed image is a projection of the sample’s 3D structure. Consequently, all features at different depths are superimposed, which can make the analysis of thick or structurally complex samples challenging. Furthermore, ptychography itself has a physical limitation on sample thickness to ensure the validity of the reconstruction. The maximum allowable thickness due to the depth of field, 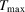, has been investigated and can be approximated by the relation 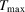 ≈ 5.2(δ*r*)^2^/λ (Tsai *et al.*, 2016 ▸), where δ*r* is the target spatial resolution and λ is the X-ray wavelength.

To meet these thickness requirements, samples are typically prepared on electron microscopy-style supports, such as metal grids or silicon nitride (SiN) membrane chips. A common support used at TPS 25A2 is an SiN membrane chip with dimensions of 5 mm × 5 mm × 0.525 mm. This chip features a 1 mm × 1 mm clear aperture covered by a 1 µm thick SiN membrane. The membrane itself is essentially transparent to hard X-rays at this energy and serves as a robust support onto which samples can be dispersed or deposited.

### 3.3. Spatial resolution benchmark

**Figure 3:**
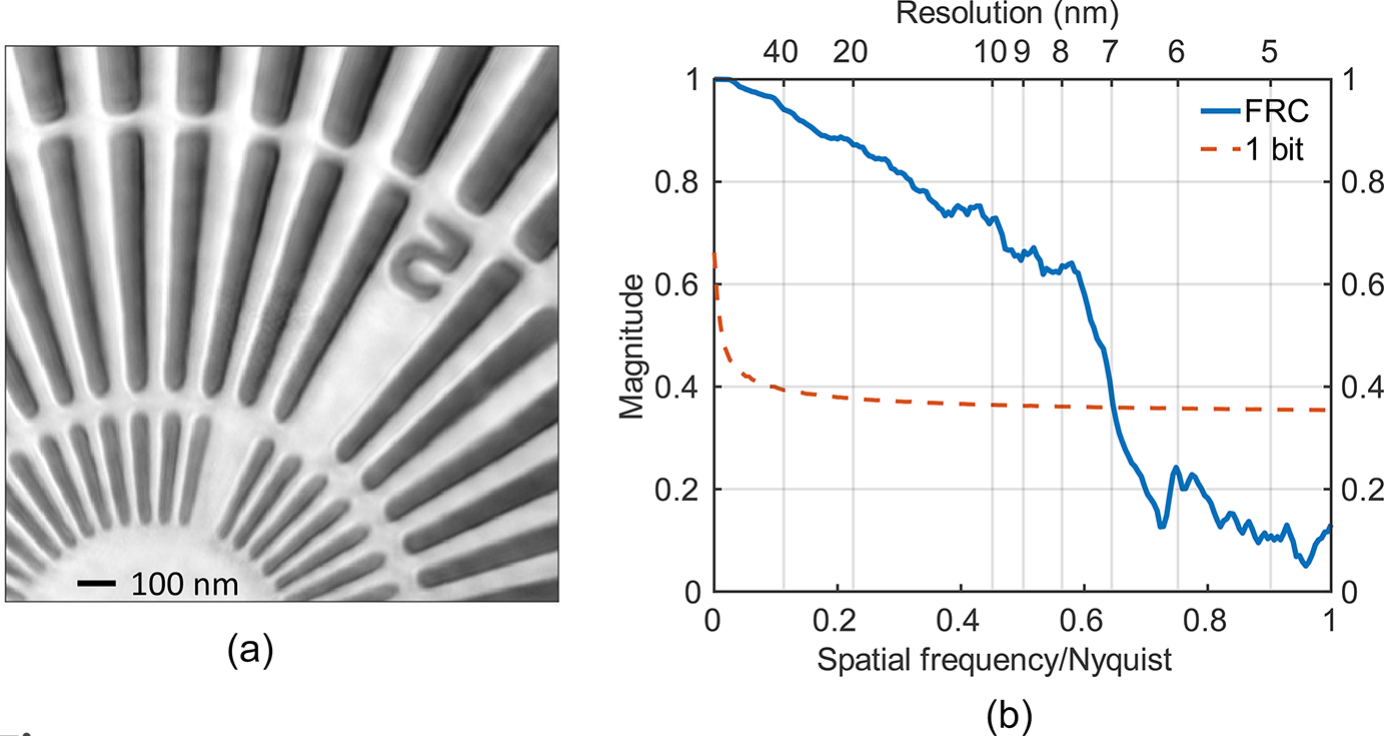
Demonstratuon of ptychography in TPS 25A2. (*a*) The reconstructed phase image of the standard sample and (*b*) the FRC result of part (*a*), showing that the spatial resolution is 6.9 nm.

To quantitatively benchmark the performance of the TPS 25A2 endstation, a standard test pattern was imaged. Fig. 3 ▸(*a*) shows the retrieved phase image of a gold Siemens star patterned on an SiN membrane (Applied Nanotools; Alberta, Canada). The structure has a height of 600 nm and features a minimum half-pitch of 25 nm.

The demonstration was performed at a photon energy of 8.979 keV using the Eiger 16M detector placed at an SDD of 3.9 m. An illuminating beam with a diameter of approximately 400 nm was produced by positioning the sample 200 µm downstream of the zone plate’s focal plane. A total of 287 diffraction patterns were collected along a Fermat-spiral scan path (Huang *et al.*, 2014 ▸), with a scan range of 1.5 µm and a step size of 50 nm. The exposure time for each pattern was 1 s.

Ptychographic reconstructions were performed using the *PyNX* software package and an in-house program. The reconstruction was performed on a 1600 × 1600 pixel array. Based on the experimental geometry, the pixel size, Δ*x*, of the reconstructed image is calculated to be 4.5 nm. A high-quality image can typically be obtained after a 200-iteration process, which takes approximately 4 min with GPU acceleration. For specific datasets requiring custom analysis, a flexible in-house program is also utilized.

The spatial resolution of the reconstructed image was determined using the Fourier ring correlation (FRC) method (van Heel & Schatz, 2005 ▸). The resulting FRC curve, shown in Fig. 3 ▸(*b*), indicates that the best spatial resolution of 6.9 nm (7.2 nm using 10–90% line-cut criteria) was achieved for this dataset.

## 4. Application showcase

As demonstrated in the previous section, the benchmark results confirm that ptychography at TPS 25A2 is a powerful hard X-ray imaging technique. To showcase its versatility beyond idealized test patterns, we present results from two distinct types of samples: inorganic nanoparticles and a complex biological structure. The experimental conditions were the same as for the measurement of the standard sample.

### 4.1. Imaging of nanoparticles in an ambient environment

**Figure 4:**
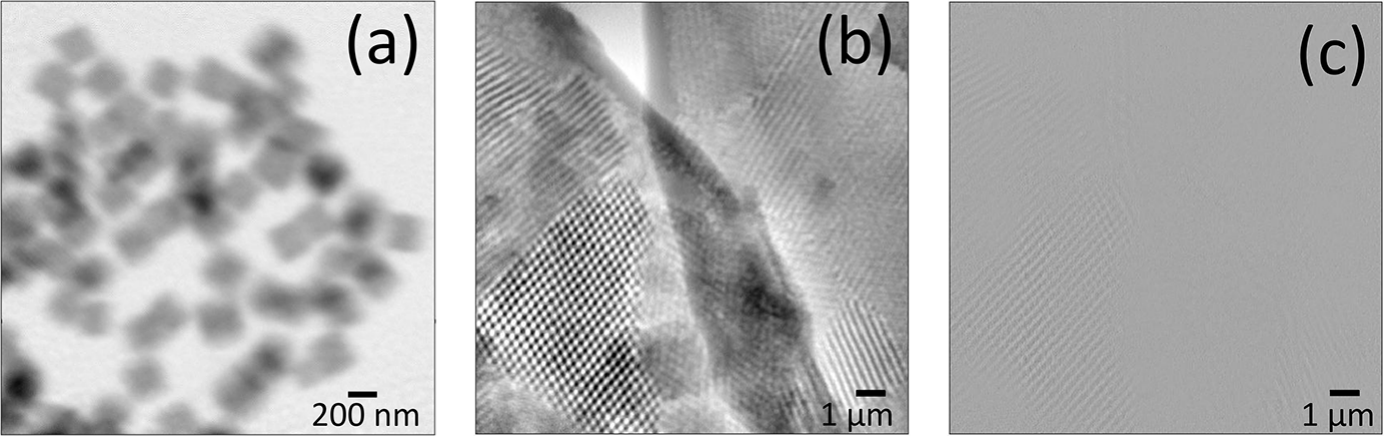
(*a*) Reconstructed phase image of Cu_2_O nanoparticles, achieving a spatial resolution of 35.5 nm. (*b*) Reconstructed phase image of a *Pachyrhynchus nobilis* scale. (*c*) Corresponding amplitude image of the same reconstruction shown in part (*b*), demonstrating the enhanced contrast available in the phase channel for low-*Z* materials.

Fig. 4 ▸(*a*) shows the retrieved phase image of Cu_2_O nano-cubics with an average size of approximately 300 nm. The data were collected at a photon energy of 8.959 keV, which is 20 eV below the Cu *K*-edge. This energy was chosen to mitigate beam-induced sample degradation. The exposure time was 1 s per frame, the beamsize was approximately 400 nm and a Fermat sprial scanning path with 287 steps was performed. For this dataset, a spatial resolution determined by FRC of approximately 35.5 nm was achieved.

Crucially, this measurement was performed in an ambient environment (at room temperature and atmospheric pressure). This result not only demonstrates the capability to image isolated weakly scattering nanoparticles, but also highlights the flexibility of the sample environment, underscoring the endstation’s potential for future *in situ* and operando high-resolution studies.

### 4.2. Application to a low-*Z* complex structure

To demonstrate the endstation’s performance on a complex low-*Z* biological sample, we imaged the scales of the *Pachyrhynchus nobilis* (Chang *et al.*, 2020 ▸). The vibrant structural color of this species is known to originate from an internal complex photonic crystal structure. With structural features such as chitin struts measuring approximately 58 nm in radius, this intricate natural nanostructure serves as an ideal sample to showcase the high-resolution capabilities of our ptychography setup. The exposure time were 1 s per frame, the beamsize was approximately 400 nm and a Fermat sprial scanning path with 287 steps was performed.

The retrieved phase image, shown in Fig. 4 ▸(*b*), reveals the intricate network of the chitin structure, with fine features being clearly distinguishable. The corresponding amplitude image is shown for comparison in Fig. 4 ▸(*c*). This amplitude image pro­vides a map of the material’s X-ray transmission (or absorption). In contrast, the phase image reveals rich structural details. This comparison demonstrates that for complex low-*Z* materials such as this, the phase-contrast channel pro­vided by ptychography is particularly effective for visualizing fine morphological features.

## 5. Summary and outlook

In summary, we have successfully designed, constructed and commissioned a hard X-ray ptychography endstation, TPS 25A2, at the Taiwan Photon Source. The endstation, based on a zone-plate focusing optic, pro­vides a stable and user-accessible platform for nanoscale 2D imaging. We have demonstrated its capability to achieve a benchmark spatial resolution of 6.9 nm, with an experimental throughput that allows for the collection of a high-resolution dataset in approximately 7 min, corresponding to 415 resolution elements per second (Guizar-Sicairos *et al.*, 2014 ▸). The versatility of the endstation has been demonstrated through the successful imaging of both inorganic nanoparticles and complex low-*Z* biological structures, highlighting its potential for a broad range of applications in materials science and biophysics.

Future developments are currently underway to further enhance the capabilities of TPS 25A2. A pivotal direction is the establishment of ptychographic X-ray-computed tomography, which exploits the unique advantage of hard X-rays for non-destructive 3D imaging. Concurrently, we are implementing a fly scan data acquisition mode. This upgrade is critical to minimizing scanning overheads, thereby providing the high temporal efficiency required for both 3D tomography and large-area mapping.

The long-term vision is to establish spectro­ptychography capabilities. By acquiring ptychographic data across an element’s absorption edge, this technique will pro­vide nanoscale mapping of elemental distributions and chemical states, transforming the endstation into a powerful analytical microscope for advanced materials research.

The TPS 25A2 endstation is now open to the user community, and we anticipate it will become a powerful tool for a wide range of scientific discoveries.

## Figures and Tables

**Table 1:** Key parameters and notations in ptychography

Notation	Formula/relation	Description
*P*(**r**)		Probe function; the illuminating wavefield at the sample
*O*(**r**)		Object function; the complex transmission function of the sample
ψ(**r**)	*P*(**r**)·*O*(**r**)	Exit wave; the wavefield immediately after the sample
Ψ(**q**)	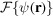	Diffraction wavefield in reciprocal space
*I*(**q**)	|Ψ(**q**)|^2^	Diffraction pattern; the recorded intensity
Δ*P*		Pixel size of the detector
SDD		Sample-to-detector distance
λ		Wavelength of the incident X-ray
*N*		Number of pixels along one dimension of the detector
Δ*x*	SDD·λ/*N*·Δ*P*	Pixel size in the reconstructed real-space image
*W*	*N*·Δ*x*	The CDI window; the field of view of the probe function and exit wave (Burdet *et al.*, 2014 ▸)
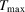	5.2(δ*r*)^2^/λ	The maximum thickness of samples due to the depth of field; δ*r* is the resolution of reconstructed images (Tsai *et al.*, 2016 ▸)

## Data Availability

The data that support the findings of this study are available from the corresponding author upon reasonable request.
